# Titan Cells and Yeast Forms of *Cryptococcus neoformans* and *Cryptococcus gattii* Are Recognized by GXMR-CAR

**DOI:** 10.3390/microorganisms9091886

**Published:** 2021-09-05

**Authors:** Matheus Henrique dos Santos, Michele Procópio Machado, Pappanaicken R. Kumaresan, Thiago Aparecido da Silva

**Affiliations:** 1 Department of Cell and Molecular Biology and Pathogenic Bioagents, Ribeirão Preto Medical School, University of São Paulo, Ribeirão Preto 140490-900, SP, Brazil; matheus.zeit@outlook.com (M.H.d.S.); michele_procopio@hotmail.com (M.P.M.); 2 Department of Lymphoma and Myeloma, The University of Texas MD Anderson Cancer Center, Houston, TX 77030, USA; pkumaresan@mdanderson.org

**Keywords:** chimeric antigen receptor, *Cryptococcus* spp., glucuronoxylomannan, cell therapy

## Abstract

Cryptococcosis, a systemic mycosis that affects both the immunocompromised and immunocompetent, is caused by the inhalation of dehydrated yeasts or fungal spores of *Cryptococcus gattii* or *Cryptococcus neoformans*. The *Cryptococcus* spp. polysaccharide capsule is composed mainly of glucuronoxylomannan—GXM, its major virulence factor. The capsule thickness increases to more than 15 μm during titanization, favoring the pathogenesis of cryptococcosis. Previous studies demonstrated that cytotoxic T cells that had been bioengineered with GXM-targeting chimeric antigen receptor (GXMR-CAR) were able to recognize *C. neoformans* by promoting the control of titanization. GXMR-CAR, a second-generation CAR, contains a single-chain variable fragment that originates from a 18B7 clone: a human IgG4 hinge, followed by a human CD28 (transmembrane/cytoplasmic domains) and a CD3ς chain. In the current study, we redirected T cells to target distinct *C. neoformans* and *C. gattii* cell types by GXMR-CAR. Lentiviral particles carrying the GXMR-CAR sequence were used to transduce Jurkat cells, and these modified cells interacted with the GXM of the *C. gattii* R265 strain. Moreover, GXMR-CAR mediated the recognition of *C. gattii* and *C. neoformans* yeasts with both thin and thick polysaccharide capsules, and GXMR-CAR Jurkat cells interacted with titan cells sourced from both *Cryptococcus* spp. Thus, bioengineered cells using CAR can improve the treatment of cryptococcosis.

## 1. Introduction

Cryptococcosis is a systemic mycosis caused by the inhalation of dehydrated yeasts or fungal spores of *Cryptococcus* spp., which enter the lungs and reach the alveoli [1]; its global incidence is estimated to be approximately 220,000 cases annually of *Cryptococcus neoformans* infection, with 181,000 deaths [2]. The *Cryptococcus* genus contains more than 30 species, but only *Cryptococcus gattii* and *Cryptococcus neoformans* are clinically relevant because of their ability to affect both immunocompetent and immunocompromised individuals, respectively [3]. In immunocompromised hosts, cryptococcosis can lead to pneumonia. Latent infection with *Cryptococcus* spp. can present in otherwise immunocompetent individuals, and immunosuppression-related events may reactivate the infection [4]. *C. neoformans* has a predilection for the central nervous system, whereas *C. gattii* is detected in the majority of pulmonary tissue [5].

*Cryptococcus* spp.’s polysaccharide capsule, which is composed mainly of glucuronoxylomannan (GXM) (90–95%), glucuroxylomanogalactan (5–8%), and mannoproteins (1%) [6], surrounds the fungal cell body and is the major virulence factor [7]. The capsule’s polysaccharide composition allows the pathogen to resist dehydration, inhibit phagocytosis, and protect the cells against reactive oxygen species released by the host [8]. GXM has a molecular mass of 1700–7000 kDa [6,9], and all serotypes are composed of a linear main-chain of α-(1,3)-mannan, with β-(1,2)-glucuronic acid residues linked to the first mannose, forming a basic core. Mannosyl residues can be 6-O-acetylated and replaced by xylose units in β-(1,2) or β-(1,4), resulting in different serotypes. The xylose, mannose, glucuronic acid, and mannose residues are found in different molar ratios depending on the serotype [6]. The structure of *C. gattii* GXM contains an additional xylose residue compared to *C. neoformans* GXM [10].

The thickness of the polysaccharide capsule affects *Cryptococcus* spp. pathogenesis [11] and is regulated by the host–pathogen interaction and distinct environmental stimuli, such as osmolarity, pH, CO_2_ availability, nutrient concentration, and temperature [9]. *Cryptococcus* spp. yeasts grown on Sabouraud Dextrose (SD) medium have a thin and uniformly sized capsule [12]. In vitro studies have demonstrated that a thick capsule is induced by a CO_2_-rich atmosphere, low concentrations of iron, lower glucose levels, and low pH. The high concentration of iron in the brain favors a thinner capsule size [13]. Within hours of *Cryptococcus* spp. infection, the polysaccharide capsule thickness increases [12]. In addition, depending on the location within the host, the thickness of the *Cryptococcus* spp. capsule is distinct, and the capsule diameter in the lungs is larger than that in the brain [12].

*Cryptococcus* spp. yeasts usually have a diameter of 4–7 μm, and the inhalation of the yeast by the host initiates morphological changes in yeast size, with reported cell diameters of 100 μm [14,15]. Titan cells have a diameter larger than 15 μm; they contribute to virulence through resistance to stress factors and inhibition of phagocytosis [16]. These factors contribute to the pathogenesis of *Cryptococcus* spp. [15,16]. The titanization process can be controlled by cytotoxic T cells using an engineered chimeric antigen receptor (CAR) that targets GXM expressed on the cell surface of *C. neoformans*, as reported by da Silva et al. [17]. The authors demonstrated that T cells expressing a GXM-specific CAR (GXMR-CAR) containing a single-chain variable fragment from a 18B7 clone were able to target GXM, decreasing the percentage of titan cells at the *C. neoformans* infection site.

The present study determined whether GXMR-CAR, a second-generation CAR that contains a modified human IgG4 hinge, followed by a human CD28 (transmembrane and cytoplasmic domains) and a CD3ς chain [17], could redirect T cells to target distinct *C. neoformans* and *C. gattii* cell types in a Jurkat cell line. We demonstrated that *Cryptococcus* spp. with distinct polysaccharide capsule thicknesses and even titan cells are recognized by GXMR-CAR T cells. These data reinforce the use of CAR T cells to target *Cryptococcus* spp.

## 2. Materials and Methods

### 2.1. Heat-Killed C. gattii and C. neoformans Cells

The *C. gattii* R265 (VGII molecular genotype) and *C. neoformans* H99 (serotype A) yeast strains were recovered from −80 °C (20% glycerol stock; Sigma-Aldrich, Burlington, MA, USA) and grown overnight in SD broth or yeast nitrogen base (YNB) medium at 30 °C with continuous agitation (150 rpm) for 20 h. The cells were harvested and washed in phosphate-buffered saline (PBS; Thermo Fisher Scientific, Waltham, MA, USA), and *C. gattii* and *C. neoformans* were resuspended in PBS. These cells were heat-killed and inactivated at 70 °C for 50 min, and the yeast concentrations were determined using a Neubauer chamber (Precicolor HBG, Giessen-Lützellinden, Germany).

### 2.2. Generation of Cryptococcus *spp.* Titan Cells

Titan cells were generated as reported by Trevijano-Contador et al. [15,18]. In brief, yeasts were grown for 24 h in SD broth, and 5 × 10^4^/mL cells were inoculated in 6-well plates containing titan cell medium, which is composed of 5% SD and 5% fetal bovine serum diluted in 50 mM of 3-(*N*-morpholino)propanesulfonic acid (pH of 7.3) and 15 µM of sodium azide. The yeasts were maintained at 37 °C in a 5% CO_2_ atmosphere for 24 h, and the formation of titan cells was determined by optical microscopy (Nikon, Tokyo, Japan).

### 2.3. Construction of CAR Targeting Cryptococcus *spp.*, GXMR-CAR

The design and construction of GXMR-CAR to target *Cryptococcus* spp. was reported by da Silva et al. [17]. The GXM-binding domain localized in the extracellular portion consists of a single-chain variable fragment (scFv) of the anti-GXM mAb 18B7 clone, which was generated by Casadevall et al. [19]. GXMR-CAR has a modified human IgG4 hinge and Fc regions, followed by CD28 transmembrane and co-stimulatory domains and the signaling domain of CD3ζ.

### 2.4. Lentiviral Particle Production

GXMR-CAR cloned into a lentiviral vector (Addgene; cat. no. 61422) was used to transfect HEK-293T cells, and pMD2.G (VSV-G envelope-expressing plasmid cat. no. 12259; Addgene, Watertown, MA, USA) and psPAX2 (second-generation lentiviral packaging plasmid cat. no. 12260; Addgene, Watertown, MA, USA) accessory plasmids were also considered for transfection of HEK-293T cells (1 × 10^6^/well) in 6-well plates with these plasmids using Lipofectamine (Thermo Fischer Scientific, Waltham, MA, USA), according to the manufacturer’s instructions. Cell supernatant containing GXMR-CAR^+^ viral particles was collected 24, 48, and 72 h after transfection, and the pool of lentiviral particles was centrifuged (300× *g* at room temperature for 10 min) and filtrated (polyvinylidene difluoride syringe filter, 0.45 µm; Kasvi, São José dos Pinhais, Brazil). Aliquots were frozen at −80 °C.

### 2.5. Generation of GXMR-CAR Jurkat Cells by Transduction

**Figure 1:**
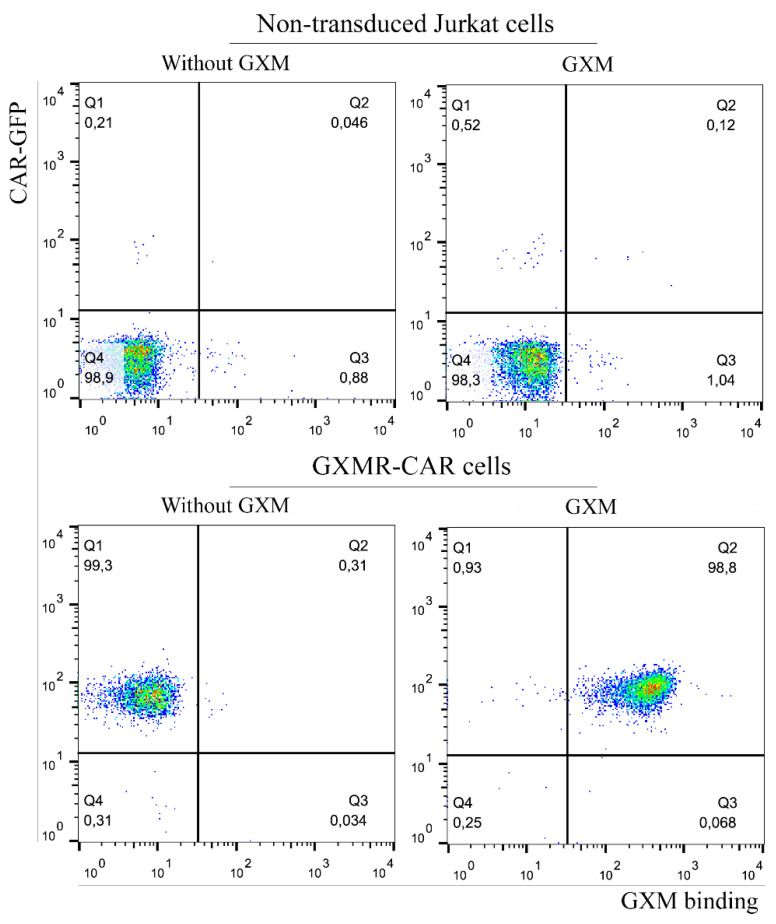
Interaction of GXMR-CAR cells with soluble GXM from *C. gattii* R265. A total of 5 × 10⁵ cells of GXMR-CAR and non-transduced Jurkat cells were incubated with soluble GXM (200 µg/mL) obtained from *C. gattii*. The detection of cell surface GXM was performed after incubation with murine anti-GXM antibody, followed by biotin-labeled goat anti-mouse IgG and streptavidin- phycoerythrin. GFP-expressing GXMR-CAR cells are visualized on the y-axis, and GXM binding is observed on the x-axis by PE fluorophore after acquisition by flow cytometry.

Transduction of Jurkat cells to express GXMR-CAR used 5 × 10^4^ cells/well seeded in 24-well plates with lentiviral particles at 1:2 dilution, as described above. Five days after transduction, the Jurkat cells that were positive for green fluorescent protein (GFP) were sorted (BD FACSAria III) for enrichment. GXMR-CAR^+^ Jurkat cells were expanded in RPMI 1640 medium (Thermo Fisher Scientific, Waltham, MA, USA)), supplemented with 10% heat-inactivated fetal bovine serum (Corning), 1 mM sodium pyruvate (Thermo Fisher Scientific, Waltham, MA, USA), and 1% penicillin/streptomycin (Sigma-Aldrich, Burlington, MA, USA). Isolation of a homogeneous cell population that expressed GXMR-CAR on the cell surface was performed by limiting dilution in 96-well plates, as reported by Greenfield [20]. For this, starting from a solution of 8 cells/mL, 100 µL/well were seeded in 96-well plates; after 2 days, the cells were fed with 100 µL of RPMI 1640 medium. Every 2 days, 100 µL of RPMI 1640 medium were replaced with fresh medium, and the entire GFP-expressing cell population was evaluated for cell surface GXMR-CAR expression by detection of soluble GXM using flow cytometry, as described in the next subsection. A homogeneous GXMR-CAR-expressing cell population was isolated, and almost the entire cell population had GXMR-CAR on cell surface expression, as shown in Figure 1B (98.8% of double-positive cells for GFP and PE).

### 2.6. Detection of GXMR-CAR on the Cell Surface by Flow Cytometry

Soluble GXM was obtained from *C. gattii*, as reported by Wozniak and Levitz [21], at a concentration of 200 µg/mL; it was incubated with GXMR-CAR Jurkat cells (1 × 10^6^ cells/mL) for 40 min. Cells were washed to remove unbound GXM, and murine anti-GXM monoclonal antibody (18B7 clone; Merck) was incubated with GXMR-CAR Jurkat cells for 45 min on ice. The cells were washed with cold PBS and incubated with goat anti-mouse IgG biotin-conjugated secondary antibody for 45 min. After the cells were washed, streptavidin-conjugated phycoerythrin (PE) (Thermo Fisher Scientific, Waltham, MA, USA) was added, and after 30 min, the cells were washed and analyzed using flow cytometry (Millipore Guava Easycyte Mini, Burlington, MA, USA). All the above steps were also performed with non-transduced Jurkat cells to demonstrate the absence of non-specific GXM binding. The data obtained were analyzed using the FlowJo™ software (version 10, for Windows; Ashland, OR, USA: Becton, Dickinson and Company; 2019).

### 2.7. Interaction of GXMR-CAR^+^ Cells and Cryptococcus *spp.* by Fluorescence Microscopy

The heat-killed *C. gattii* or *C. neoformans* cells (5 × 10^5^ cells/mL) generated from yeast or titan cells were labeled with Calcuofluor-white (Sigma-Aldrich, Burlington, MA, USA, cat. no. 18909) at a concentration of 100 µg/mL for 30 min. Yeast or titan cell forms were incubated with GXMR-CAR^+^ Jurkat cells or non-transduced Jurkat cells at a concentration of 5 × 10^5^ cells/mL in 96-well plates, and the GXMR-CAR:*Cryptococcus* spp. ratio was 1:1. The co-culture was incubated for 5 h at 37 °C, and fluorescence microscopy (Leica MI400B, Wetzar, Germany) was used to visualize the interaction between GXMR-CAR cells (GFP, green) and yeast or titan cell forms (Calcofluor, blue) at 200× magnification.

## 3. Results

### 3.1. GXMR-CAR Interacts with Soluble GXM of C. gattii R265

The major virulence factor of *Cryptococcus* spp. is a polysaccharide capsule that confers protection against the host immune system, and GXM is a major cryptococcal capsule component. This polysaccharide results in small differences in the monosaccharide composition of *Cryptococcus* spp. In the current study, we evaluated the capacity of GXMR-CAR to bind to the GXM of *C. gattii* R265; as reported by da Silva et al., GXMR-CAR interacts with GXM of *C. neoformans* H99 [17]. To investigate the recognition of the GXM in *C. gattii* R265 by GXMR-CAR, lentiviral particles carrying the GXMR-CAR sequence were used to transduce Jurkat cells, generating a stable GXMR-CAR Jurkat cell line. The GXM of *C. gattii* R265 was incubated with the GXMR-CAR Jurkat cells, and the interaction between GXM and GXMR-CAR was detected using an approach that was previously reported by da Silva et al. [17], which is presented in Figure 1. GFP-labeled GXMR-CAR Jurkat cells interacted with soluble GXM, as detected by murine anti-GXM antibody, followed by biotin-labeled goat anti-mouse IgG and streptavidin-PE. The cells stained with GFP and PE (double-positive) demonstrated recognition of soluble GXM by GXMR-CAR, whereas non-transduced Jurkat cells did not bind to soluble GXM (Figure 1).

### 3.2. GXMR-Car Redirects Jurkat Cells to Recognize the Yeast Form of C. neoformans and C. gattii

Considering that GXMR-CAR Jurkat cells bind to soluble GXM, we investigated the redirection of Jurkat cells by GXMR-CAR to target the yeast form of *C. gattii* and *C. neoformans*. Moreover, the effect of the thickness of the capsule of *Cryptococcus* spp. in the recognition by GXMR-CAR was also evaluated using *C. gattii* and *C. neoformans* grown in SD or YNB medium, which yielded thin and thick capsules, respectively. The yeasts in a co-culture with GXMR-CAR Jurkat or non-transduced cells were heat-killed and stained with Calcofluor-white, and their interaction was analyzed by fluorescence microscopy.

**Figure 2:**
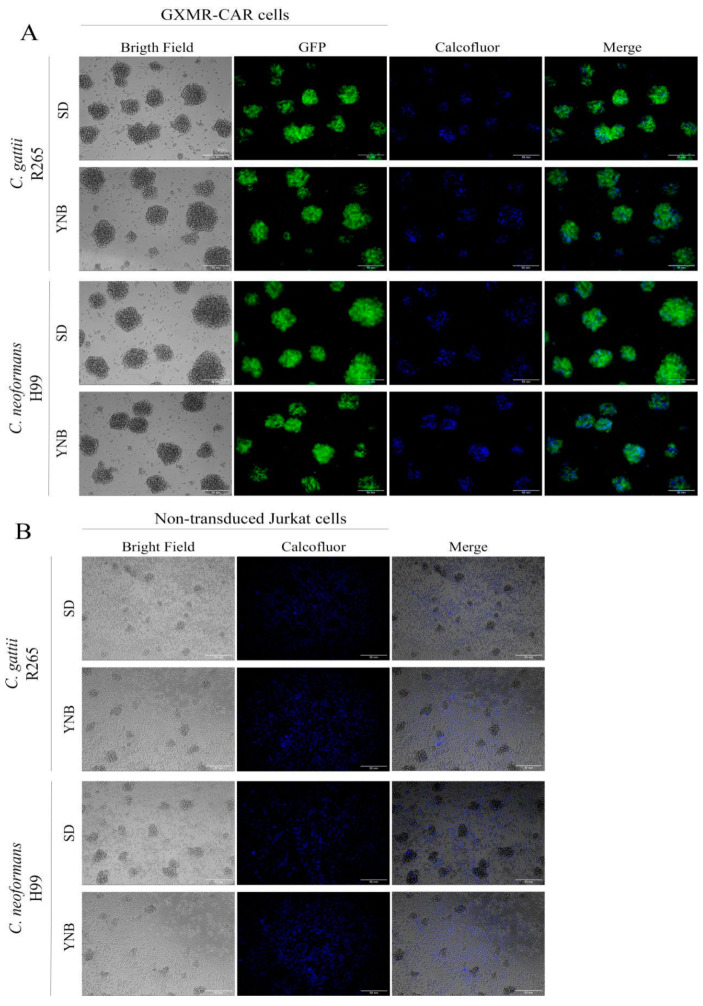
In vitro recognition of *C. gattii* and *C. neoformans* by GXMR-CAR cells. GXMR-CAR and non-transduced Jurkat cells were incubated with heat-killed *C. gattii* R265 or *C. neoformans* H99 yeasts grown in sabouraud dextrose (SD; thin capsule) or yeast nitrogen base (YNB; thick capsule) medium. Yeasts were labeled with Calcofluor-white and incubated with the cells for 5 h in a 1:1 ratio; the interaction was evaluated using fluorescence microscopy. The images were acquired at 200× magnification. (**A**) GXMR-CAR cells and yeasts (bright field), GXMR-CAR cells (green, GFP), yeasts (blue, calcofluor), and their interaction (merge). (**B**) Non-transduced Jurkat cells and yeasts (bright field), and yeasts (blue, calcofluor), and their interaction (merge).

As shown in Figure 2A, GXMR-CAR Jurkat cells interacted with *C. gattii* R265 and *C. neoformans* H99, which was demonstrated by the aggregation between GXMR-CAR Jurkat cells (GFP, green) and yeast forms (Calcofluor, blue). Moreover, GXMR-CAR Jurkat cells interacted with the yeast form grown in SD or YNB medium, whereas the non-transduced cells did not induce the aggregation of yeasts into clusters of Jurkat cells (Figure 2A,B). Then, *Cryptococcus* spp. yeasts are colocalized with the clusters of GXMR-CAR Jurkat cells, whereas most of the yeasts are around the non-transduced Jurkat cells (Figure 2). These findings demonstrate that GXMR-CAR allows the redirection of Jurkat cells to target *Cryptococcus* spp. with distinct capsule sizes.

### 3.3. Cryptococcus *spp.* Titan Cells Are Targeted by GXMR-CAR

**Figure 3:**
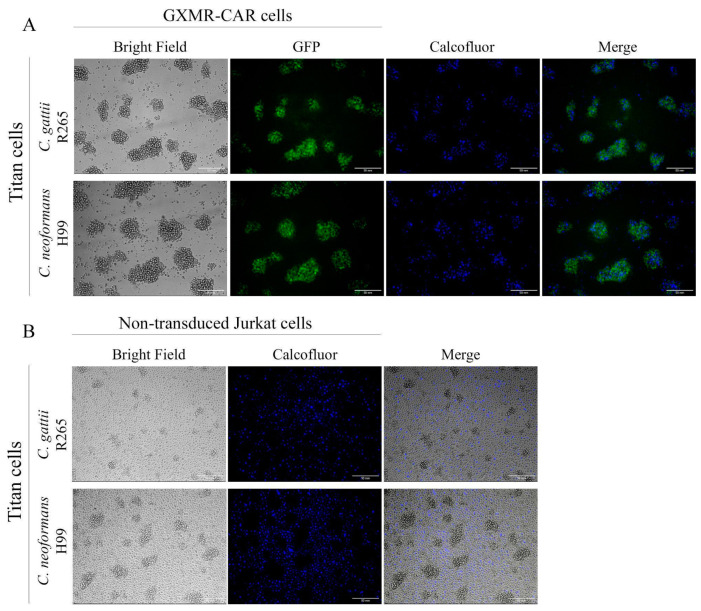
In vitro recognition of *Cryptococcus* spp. titan cells by GXMR-CAR. *C. gattii* R265 and *C. neoformans* H99 cells were grown overnight in a titan cell medium to generate titan cells. The yeasts were heat-killed and labeled with calcofluor, and GXMR-CAR or non-transduced Jurkat cells were co-cultured with titan cells for 5 h in a 1:1 ratio. The interaction was visualized in fluorescence microscopy, and the images were acquired at 200× magnification. (**A**) GXMR-CAR cells and titan cells (bright field), GXMR-CAR cells (green, GFP), and titan cells (blue, calcofluor), and their interaction (merge). (**B**) Non-transduced Jurkat cells and titan cells (bright field), titan cells (blue, calcofluor), and their interaction (merge).

The *Cryptococcus* spp. are able to produce titan cells that are useful for escaping host immune response, which is a major concern in the treatment of cryptococcosis. Titan cell formation is an important virulence factor that inhibits the activity of phagocytes. The current study evaluated the capacity of GXMR-CAR to redirect T cells to target titan cells. Titan cells were formed using the TCM (Titan cell medium), as described above, and heat-killed titan cells of *C. gattii* and *C. neoformans* were stained with Calcofluor-white before incubation with GXMR-CAR Jurkat cells or non-transduced Jurkat cells. The aggregation between GXMR-CAR Jurkat cells and titan cells was demonstrated using fluorescence microscopy, and GXMR-CAR redirected Jurkat cells to interact with titan cells sourced from both *Cryptococcus* spp. studied (Figure 3A). These data are supported by the absence of aggregation between non-transduced Jurkat cells and titan cells, as evidenced by cell clusters surrounded by *Cryptococcus* spp. (Figure 3B). These results, taken together, demonstrate a strong capacity of GXMR-CAR to redirect Jurkat cells to target GXM localized in distinct *C. gattii* and *C. neoformans* cell types.

## 4. Discussion

CAR T cell therapy is a promising option for the treatment of infectious diseases and invasive fungal infections [17,22,23,24,25,26]. GXMR-CAR was found to interact with GXM of the capsule of *Cryptococcus* spp., and the biological activity of GXMR against *C. neoformans* was also investigated [17]. The capsule polysaccharide of *C. neoformans* has small differences in the composition of monosaccharide compared to *C. gattii*, which reinforce the need to evaluate the recognition of GXM of the capsule of *Cryptococcus* spp. by GXMR-CAR. In addition, the capsule thickness and titan cells and yeast forms of *Cryptococcus* spp. present clinical relevance, as previously reported that the growth of the polysaccharide capsule was found to be associated with increased intracranial pressure in cases of cryptococcal meningitis [27]. In the current study, we assessed the capacity of GXMR-CAR to target GXM expressed in titan cells and yeast forms of *C. gattii* (R265) and *C. neoformans* (H99). Jurkat cells were modified by lentiviral transduction to express GXMR-CAR, and the yeast forms of R265 and H99 strains with a distinct polysaccharide capsule thickness were recognized by GXMR-CAR as well as by soluble GXM (Figure 1 and Figure 2). Interestingly, the single-chain variable fragment domain of GXMR-CAR redirected Jurkat cells to interact with titan cells generated from *C. gattii* and *C. neoformans* (Figure 3), indicating that GXM is targeted by GXMR-CAR under distinct conditions.

The murine monoclonal antibody 18B7 was initially developed to target GXM from *C. neoformans* [19]; GXM has considerable heterogeneity, as seen in the structural variations that are used to classify *Cryptococcus* serotypes [28]. The variations in the GXM structure depend on the position of the xylose residues and O-acetyl groups that are attached to the α-(1-3) mannan backbone [29]; in addition, environmental conditions can influence the pattern of modifications in GXM. Urai et al. [10] reported that differences in the *O*-acetylation of GXM decreased the affinity of anti-GXM antibodies. Moreover, isolated GXM fractions from the hypervirulent *C. gattii* JP02 strain induced lower levels of pro-inflammatory cytokines in dendritic cells than did those from the *C. neoformans* H99 strain, and the GXM of the JP02 strain was poorly recognized by dendritic cells [10]. These findings support the importance of GXM recognition in initiating an effector immune response over time during *Cryptococcus* spp. infection. In the current study, we demonstrated that GXMR-CAR Jurkat cells recognize soluble GXM from *C. gattii* R265 (Figure 1), and a previous study reported the capacity of GXMR-CAR to bind to *C. neoformans* H99 [17]. These data reinforce the recognition of GXM from distinct sources by GXMR-CAR; further studies should investigate the interaction between GXMR-CAR and GXM purified from clinical isolates of *Cryptococcus* spp.

The *Cryptococcus* spp. capsule is dynamic, which we accounted for in our evaluation of GXMR-CAR’s ability to recognize *Cryptococcus* spp. yeast with different polysaccharide capsule thicknesses. The capsule is the major virulence factor of *Cryptococcus* spp., and its thickness can undergo rearrangement during cryptococcosis, in which the *Cryptococcus* spp. changes the capsule composition and thickness in a microenvironment-dependent manner [12]. In this context, microenvironmental conditions can cause chemical changes in the GXM molecule, impairing the recognition of yeasts by the host immunity receptors. In the current work, we cultured *C. gattii* R265 and *C. neoformans* H99 strains in YNB and SD medium, which resulted in thick and thin capsules, respectively. Our results demonstrated that GXMR-CAR Jurkat cells were able to target *C. gattii* and *C. neoformans* grown in YNB and SD medium. These data reinforce the affinity of GXMR-CAR to the capsule polysaccharide of *Cryptococcus* spp., leading to new perspectives on the development of GXMR-CAR with distinct hinge/transmembrane domains to evaluate its impact on the recognition of *Cryptococcus* spp. The evaluation of GXMR-CAR with distinct hinge/transmembrane domains should be considered because of the predilection for the CD8 molecule as the hinge/transmembrane portion [17]. An intracellular domain could be investigated to optimize GXMR-CAR T cell function. These factors are required to develop immunotherapy using adoptive T cells to treat invasive fungal infections.

The process of titanization of *Cryptococcus* spp. makes it challenging to treat cryptococcosis using antifungal drugs or immunotherapy approaches. Thus, GXMR-CAR should be able to redirect modified Jurkat cells to target titan *C. gattii* R265 and *C. neoformans* H99 cells; in the current study, we demonstrated that Jurkat cells expressing GXMR-CAR bind to the titanized form of fungi. The in vitro process of titanization of *Cryptococcus* spp. was established by Trevijano-Contador et al. [18], and a fluorescence microscopy analysis showed that the interaction between GXMR-CAR Jurkat cells and the yeast form was more pronounced than those incubated with titan cells. Several studies have described some structural differences between the capsules of yeast and titan forms [30,31] that changed the epitope presentation on the surface of yeast, compromising recognition by mAbs [4]. This behavior is associated with the inhibition of phagocytosis by antigen-presenting cells, which contributes to the progression and persistence of cryptococcosis [32,33]. Silva and collaborators [17] demonstrated that GXMR-CAR T cells decreased the size and number of titan cells in the lungs of mice infected with *C. neoformans*, demonstrating the potential protection mediated by GXMR-CAR T cells against cryptococcosis. Further studies are focused on the redirection of T cells to the lungs by GXMR-CAR over time during the *C. gattii* and *C. neoformans* infection, and also novel GXMR-CAR composed of the distinct hinge/transmembrane and signal transduction domains will be considered.

## 5. Conclusions

In conclusion, our findings demonstrate the ability of GXMR-CAR to redirect T cells to target the *C. gattii* R265 yeast as well as the titan cells from both *C. gattii* R265 and *C. neoformans* H99, which reveal an advantage of CAR T cell therapy against cryptococcosis. 

## Data Availability

The study did not report any data.
